# Racial differences in serological markers across the first year of injury in spinal cord injury: a retrospective analysis of a multi-center interventional study

**DOI:** 10.1038/s41393-024-00998-3

**Published:** 2024-07-03

**Authors:** Jia Li, Matthew Farrow, Kerollos Ibrahim, Dana M. McTigue, John Kramer, Bobo Tong, Catherine Jutzeler, Linda Jones, Ceren Yarar-Fisher

**Affiliations:** 1 College of Medicine, Department of Physical Medicine & Rehabilitation, The Ohio State University, Columbus, OH USA; 2 College of Medicine, Florida State University, Tallahassee, FL USA; 3 College of Medicine - Department of Neuroscience, The Ohio State University, Columbus, OH USA; 4 International Collaboration on Repair Discoveries (ICORD), University of British Columbia, Vancouver, BC Canada; 5 Department of Health Sciences and Technology, ETH Zurich, Zurich, Switzerland; 6 Department of Physical Therapy, Thomas Jefferson University, Philadelphia, PA USA

**Keywords:** Outcomes research, Public health

## Abstract

**Study design:**

Secondary analysis of a randomized, multi-center, placebo-controlled study(Sygen®).

**Objectives:**

To evaluate racial differences in serological markers in individuals with spinal cord injury(SCI) across the first year of injury.

**Setting:**

Hospitals in North America.

**Methods:**

Serological markers (e.g.,cell count, liver, kidney, and pancreatic function, metabolism, and muscle damage) were assessed among 316 participants (247 White, 69 Black) at admission, weeks 1, 2, 4, 8, and 52 post-injury. Linear mixed models were employed to explore the main effects of time, race (Black vs. White), and their interaction, with adjustment of covariates such as study center, polytrauma, injury (level, completeness), treatment group, and sex.

**Results:**

A main effect of race was observed where White individuals had higher alanine transaminase, blood urea nitrogen(BUN), BUN/Creatinine ratio, sodium, and chloride, while Black individuals had higher calcium, total serum protein, and platelets. For markers with interaction effects, post-hoc comparisons showed that at week 52, White individuals had higher mature neutrophils, hematocrit, hemoglobin, mean corpuscular hemoglobin, albumin, and triglycerides, and Black individuals had higher amylase. Eosinophils, monocytes, red blood cells, aspartate aminotransferase, bilirubin, cholesterol, partial thromboplastin time, urine specific gravity, urine pH, CO2, and inorganic phosphorus did not differ between races.

**Conclusions:**

Our results revealed racial differences in serological markers and underscores the importance of considering race as a determinant of physiological responses. Future studies are warranted to explore the causes and implications of these racial disparities to facilitate tailored clinical management and social policy changes that can improve health equity.

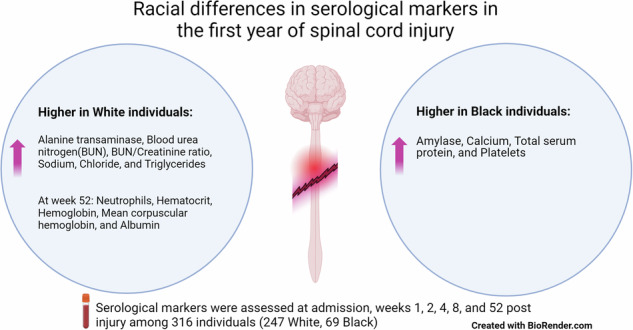

## Introduction

In 2019, the global incidence of spinal cord injury (SCI) was calculated as approximately 1 million [1]. The primary ethnic make-up of new SCIs in the U.S. are non-Hispanic Whites (56.4%), non-Hispanic Blacks (24.8%), and Hispanics (14.1%) [2]. However, the incidence of SCI is disproportionally higher in Black (35.3 cases per million) compared to White Americans (21.3 cases per million) [3]. Given the complex genetic, socioeconomic, and healthcare-related factors, Black individuals may experience significant disparity in their recovery journey.

Racial disparities in health outcomes following a SCI have been documented [4]. For example, during initial hospitalization following an SCI, Black Americans experience more complications and are less likely to be discharged into inpatient rehabilitation facilities compared to their White counterparts [5]. Additionally, Black individuals experience a higher number of rehospitalization days [6], an increased likelihood of developing pressure ulcers [6, 7], and a higher prevalence of self-reported comorbidities such as diabetes and hypertension [7].

Acute SCI profoundly impacts multiple physiological systems, leading to complex changes in blood chemistry, immune responses, and metabolic processes. Standard serological markers (e.g., cell counts, kidney, liver, and pancreatic function), which could serve as valuable indicators of systemic health and physiological processes, are typically at pathological concentrations in the first two weeks post-injury before returning to normal values within 6–12 months post-injury [8]. The severity of SCI, age at injury, and sex were found to influence these responses [8]. However, racial differences in these serological markers have not been evaluated in the first year of SCI, despite some racial differences being documented in the non-SCI general population [9, 10]. Such racial disparity may stem either from 1) normal biological diversity, where different sets of references have been suggested for Black individuals for some markers (e.g., white blood count, hemoglobin) to avoid misdiagnosis and overtreatment [10], or 2) health disparities as a result of the complex interplay of social determinants of health (e.g., blood glucose) [11, 12], which would necessitate tailored clinical management and significant societal efforts to promote health equity.

Identifying racial differences in serological profiles in individuals with SCI represents a crucial first step that can facilitate the exploration of underlying mechanisms (e.g., physiological, environmental, or social) and their long-term health implications. This study aimed to determine if such differences are present after SCI as they may contribute to increased disease risk at the chronic stages of SCI. To achieve this, racial differences in serological markers across the first year of injury amongst individuals with SCI were evaluated, controlling for sex, age at injury, and severity and level of injury.

## Methods

### Study design

This study constitutes a secondary analysis of data collected from a completed prospective phase III, FDA-regulated multi-site study (Sygen^®^) assessing the efficacy of gangliosidosis-1 (GM-1) ganglioside therapy in acute traumatic SCI. Details about study design and recruitment criteria have been described and published previously [13]. Briefly, a randomized, placebo-controlled prospective study was conducted at 28 neurotrauma centers in North America across 5 years between 1992 and 1997. The study recruited patients with an injury rostral to the T10 bony level, resulting in at least one lower extremity with an American Spinal Injury Association (AIS) motor score of less than 15 of 25. The study enrolled a total of 760 eligible participants. The primary level of injury was cervical (*n* = 579, AIS A-D), and motor vehicle accident was the primary cause (*n* = 411). Participants had blood samples taken at admission to the trauma center (week 0 herein) and weeks 1, 2, 4, 8, and 52. Participants in this clinical trial received one of three treatments: placebo (loading dose of placebo and then 56 days of placebo), low-dose of GM-1 (300 mg loading dose, followed by 100 mg/day for 56 days), or high-dose of GM-1 (600 mg loading dose, followed by 200 mg/day for 56 days).

For this analysis, we only included participants with complete information on age, sex, injury information (degree of polytrauma, severity and level of injury), and race. Additionally, we only included participants for which a complete set of serological data at all time points was available. Due to the limited number of participants of another race, only racial differences between White and Black individuals were evaluated.

### Outcome assessment

A total of 38 blood markers, including complete blood count (white blood cells, mature neutrophils, eosinophils, monocytes, lymphocytes, basophils, hematocrit, hemoglobin, red blood cells, mean corpuscular hemoglobin [MCH], mean corpuscular hemoglobin concentration [MCHC], mean corpuscular volume [MCV]), liver function (aspartate aminotransferase [AST], alanine transaminase [ALT], bilirubin, Alk phosphatase), kidney function (blood urea nitrogen [BUN], creatinine, BUN/creatinine, calcium, total serum protein, albumin), pancreatic function (amylase), metabolism (cholesterol, glucose, triglycerides), clotting (platelets, prothrombin time, partial thromboplastin time, urine specific gravity, urine pH), muscle health (potassium, sodium, creatine kinase), and other (chloride, CO_2_, inorganic phosphorus, uric acid) markers were assessed at the specified time points. These blood markers were assessed as part of an FDA requirement for the study. Normal ranges for each outcome are presented in Table 1. The blood analyses were performed by SmithKline Beecham [8].

**Table 1:** List of serological markers assessed in the current study.

Outcome	Units	Reference range
Complete blood count		
White blood cells	/mm^3^	4.4–11 x 10^3^
Neutrophils	%	40–60
Eosinophils	%	0–6
Monocytes	%	2–10
Lymphocytes	%	20–40
Basophils	%	0–1
Hematocrit	%	38.8–50 (males) 34.9–44.5 (females)
Hemoglobin	g/dL	13.8–17.2 (males) 12.1–15.1 (females)
Red blood cells	mcl	4.5–5.5 (males) 4.0–5.0 (females)
Mean corpuscular hemoglobin (MCH)	pg/cell	27–31
Mean corpuscular hemoglobin concentration (MCHC)	%	32–36
Mean corpuscular volume (MCV)	μm^3^	80–100
Liver		
Aspartate transaminase (AST)	U/L	10–40
Alanine aminotransferase (ALT)	U/L	7–56
Bilirubin	mg/dL	0.3–1.2
Alk phosphatase	U/L	44–147
Pancreas		
Amylase	U/L	30–110
Kidney		
Blood urea nitrogen (BUN)	mg/dL	7–20
Creatinine	mg/dL	0.6–1.3
BUN/creatinine ratio		10–20
Calcium	mg/dL	8.5–10.5
Total serum protein (TSP)	g/dL	6.0–8.3
Albumin	g/dL	3.5–5.0
Metabolism		
Cholesterol	mg/dL	<200
Triglycerides	mg/dL	<150
Glucose	mg/dL	70–100
Muscle		
Potassium	mEq/L	3.5–5.1
Sodium	mEq/L	135–145
Creatine kinase	u/L	55–170 (males) 30–135 (females)
Clotting		
Platelets	/mm^3^	150,000–450,000
Prothrombin time	seconds	11–13
Partial thromboplastin time	seconds	25–35
Urine specific gravity	sg	1.005–1.030
Urine pH	pH	4.6–8.0
Other		
Chloride	mEq/L	98–107
CO_2_	mmHg	23–29
Inorganic phosphorus	mg/dL	2.5–4.5
Uric Acid	mg/dL	3.5–7.2

Data obtained from UpToDate (https://www.uptodate.com/contents/laboratory-test-reference-ranges-in-adults).

Injury completeness was assessed according to the American Spinal Injury Association (ASIA) Impairment Scale (AIS). Polytrauma was defined as significant injuries (three or more points), in two or more regions, in addition to SCI as previously defined [13].

### Statistical analysis

Linear mixed models were employed to explore the primary effects of time, race, and their interaction. Statistical analysis software (SAS 9.4, Cary, North Carolina) was used to perform the analyses. Participants were considered a random effect, and various covariates were controlled, including the study center, the presence of polytrauma, injury level, treatment group (GM-1 group), completeness of injury, and sex. Comparisons between races at each time point were performed using post-hoc Bonferroni adjustment. Model assumptions were evaluated, and log transformations were performed when applicable, such as in cases of non-normal distribution of residuals. Data presented are least-square means and 95% confidence intervals in the original scale.

## Results

A total of 316 participants are included in the current analysis, and the demographic and injury distribution are presented in Table 2. Briefly, the participants consisted of predominately of males (80%), cervical level injuries (71.5%), AIS classification A (63%), and absence of polytrauma (75.6%). The proportion of AIS B, C, and D classifications were 17%, 17%, and 3%, respectively.

**Table 2:** Participants characteristics.

	White (*n* = 247)		Black (*n* = 69)	
Age, y^a^	30 (21,40)		26 (20,39)	
Gender	Male	196	Male	57
	Female	51	Female	12
Severity of Injury				
C1-4 AIS^b^ A, B, C	43		25	
C5-8 AIS A, B, C	121		29	
T1-S5 AIS A, B, C	74		15	
AIS D at any injury level	9		0	
Presence of Polytrauma^c^	No	182	No	57
	Yes	65	Yes	12
BMI^d^, kg/m^2 a^	24.4 (22.1,27.7)		24.6 (22.4,27.7)	

^a^Data are median and interquartile range.

^b^The American Spinal Injury Association Impairment Scale.

^c^Polytrauma was defined as significant injuries of three or more points in two or more different anatomic regions in addition to the spinal cord injury.

^d^Body Mass Index.

### Serological markers

Values for all serological markers at each time point for each race group are presented in Table 3 and a summary of the results is presented in Table 4. There were main effects of time for all variables except urine specific gravity.

**Table 3:** Racial differences in changes in serological makers within the first year of spinal cord injury.

Outcome		Week 0	Week 1	Week 2	Week 4	Week 8	Week 52	P^a^_time_	P^a^_race_	P^a^_interaction_
Complete blood count										
White blood cells, /mm^3^	W^c^	11.9 (10.9,13)	10.9 (10,11.9)	9 (8.2,9.8)	7.3 (6.7,7.9)	6.5 (6,7.1)	6.6 (6,7.2)	**<0.01**	0.18	**<0.01**
	B	12.1 (10.7,13.8)	12.4 (10.9,14.1)	10.5 (9.3,12.0)^b^	7.8 (6.9,8.9)	7.1 (6.2,8.1)	5.9 (5.2,6.7)			
Neutrophils, %	W	84 (82,86)	74 (72,76)	73 (71,75)	64 (62,66)	60 (58,62)	59 (57,61)	**<0.01**	0.16	**<0.01**
	B	84 (81,87)	72 (69,75)	73 (70,76)	63 (60,66)	59 (56,62)	51 (48,54)^b^			
Eosinophils, %	W	0.3 (0.2,0.5)	1.4 (1.1,1.7)	1.7 (1.4,2.1)	2.5 (2.1,3.0)	2.7 (2.2,3.1)	3 (2.6,3.5)	**<0.01**	0.60	0.70
	B	0.3 (0.1,0.5)	1.1 (0.8,1.5)	1.7 (1.2,2.2)	2.6 (2.0,3.3)	2.5 (1.9,3.2)	3.2 (2.6,3.9)			
Monocytes, %	W	2.6 (2.2,2.9)	4.2 (3.7,4.7)	3.6 (3.2,4.1)	4.8 (4.2,5.4)	4.9 (4.3,5.4)	5.2 (4.6,5.7)	**<0.01**	0.11	0.11
	B	2.3 (1.9,2.9)	5.4 (4.5,6.4)^b^	4.1 (3.4,5.0)	4.9 (4.1,5.8)	5.3 (4.5,6.3)	5.7 (4.8,6.6)			
Lymphocytes, %	W	8.6 (7.2,9.9)	15.9 (14.4,17.4)	17.8 (16.3,19.4)	25 (23.3,26.6)	29 (27.3,30.7)	29.9 (28.2,31.5)	**<0.01**	0.20	0.07
	B	8.7 (6.8,10.5)	16.1 (13.9,18.4)	17.0 (14.7,19.4)	24.9 (22.3,27.5)	28.6 (25.9,31.3)	35.5 (32.9,38.1)^b^			
Basophils, %	W	0.01 (0.01,0.02)	0.06 (0.04,0.09)	0.13 (0.09,0.19)	0.31 (0.21,0.45)	0.37 (0.26,0.55)	0.35 (0.24,0.51)	**<0.01**	0.15	**0.05**
	B	0.01 (0,0.02)	0.10 (0.05,0.19)	0.11 (0.06,0.20)	0.15 (0.08,0.28)	0.25 (0.13,0.44)	0.24 (0.13,0.44)			
Hematocrit, %	W	34.4 (33.3,35.4)	33 (31.9,34.0)	33.3 (32.2,34.3)	36.3 (35.4,37.3)	38.1 (37.1,39.1)	43.1 (42.1,44.1)	**<0.01**	0.16	**0.03**
	B	35 (33.4,36.7)	32.5 (31,34.1)	32.8 (31.3,34.3)	35.6 (34.2,37.0)	37.4 (35.9,38.8)	40.7 (39.1,42.2)^b^			
Hemoglobin, g/dL	W	11.7 (11.3,12.1)	11.2 (10.9,11.6)	11.2 (10.8,11.5)	12.1 (11.8,12.5)	12.8 (12.5,13.2)	14.5 (14.1,14.8)	**<0.01**	0.06	**0.04**
	B	11.8 (11.3,12.4)	11 (10.4,11.5)	11 (10.5,11.5)	11.8 (11.3,12.2)	12.4 (11.9,12.9)	13.6 (13.1,14.1)^b^			
Red blood cells, mcl	W	3.8 (3.6,3.9)	3.6 (3.5,3.7)	3.6 (3.5,3.7)	4 (3.9,4.1)	4.3 (4.1,4.4)	4.8 (4.6,4.9)	**<0.01**	0.78	0.11
	B	3.9 (3.7,4.1)	3.6 (3.4,3.8)	3.6 (3.4,3.8)	3.9 (3.7,4.1)	4.2 (4.0,4.4)	4.6 (4.5,4.8)			
MCH, pg/cell	W	31.2 (30.7,31.6)	31.2 (30.8,31.6)	31 (30.5,31.4)	30.6 (30.1,31.0)	30.1 (29.6,30.5)	30.5 (30.0,31.0)	**<0.01**	**0.01**	**<0.01**
	B	30.4 (29.8,31.1)^b^	30.7 (30.0,31.3)	30.6 (30.0,31.2)	30.2 (29.6,30.8)	29.6 (29.0,30.3)	29.3 (28.7,30.0)^b^			
MCHC, %	W	34.1 (33.9,34.3)	34 (33.9,34.2)	33.6 (33.4,33.8)	33.4 (33.2,33.6)	33.6 (33.4,33.8)	33.6 (33.4,33.8)	**<0.01**	0.06	0.17
	B	33.7 (33.5,34.0)	33.7 (33.4,34.0)	33.5 (33.2,33.8)	33.1 (32.8,33.4)	33.2 (32.9,33.6)	33.4 (33.1,33.8)			
MCV, μm^3^	W	91.7 (90.4,93.0)	91.8 (90.5,93.1)	92.4 (91.1,93.6)	91.7 (90.4,93)	89.6 (88.3,90.8)	90.9 (89.6,92.2)	**<0.01**	0.06	**<0.01**
	B	90.1 (88.3,91.9)	91 (89.2,92.8)	91.2 (89.5,92.9)	91.4 (89.7,93.2)	89.0 (87.3,90.8)	87.7 (85.9,89.5)^b^			
Liver										
AST, U/L	W	71 (63,80)	49 (44,55)	41 (37,45)	30 (27,33)	22 (20,25)	21 (19,23)	**<0.01**	0.99	0.42
	B	73 (60,88)	55 (46,65)	40 (34,47)	31 (26,36)	20 (18,23)	20 (18,23)			
ALT, U/L	W	46 (40,53)	79 (68,92)	79 (69,92)	52 (45,60)	29 (25,34)	21 (18,24)	**<0.01**	**0.05**	0.74
	B	40.0 (33,49)	76 (60,96)	68 (54,85)	42 (33,52)	25 (20,31)	19 (15,22)			
Bilirubin, mg/dL	W	0.64 (0.57,0.71)	0.68 (0.61,0.76)	0.52 (0.47,0.58)	0.43 (0.39,0.47)	0.38 (0.35,0.42)	0.48 (0.44,0.53)	**<0.01**	0.56	0.11
	B	0.63 (0.54,0.74)	0.60 (0.51,0.71)	0.51 (0.44,0.59)	0.44 (0.38,0.5)	0.40 (0.35,0.46)	0.44 (0.38,0.51)			
Alk phosphatase, U/L	W	64 (58,70)	114 (104,125)	146 (133,160)	143 (131,157)	133 (121,146)	117 (107,128)	**<0.01**	0.09	**0.01**
	B	68 (59,78)	101 (88,116)	128 (111,146)	127 (111,146)	117 (102,134)	115 (100,132)			
Pancreas										
Amylase, U/L	W	47 (39,55)	59 (50,70)	66 (56,79)	56 (47,66)	47 (40,56)	46 (35,54)	**<0.01**	**0.02**	**<0.01**
	B	52 (41,70)	63 (49,80)	71 (55, 90)	73 (57,93)	68 (52,85)^b^	68 (53,87)^b^			
Kidney										
BUN, mg/dL	W	14.5 (13.5,15.6)	16 (14.9,17.2)	14.8 (13.7,15.9)	11.6 (10.8,12.5)	9.7 (9.0,10.4)	10.3 (9.6,11.1)	**<0.01**	**0.03**	0.39
	B	13.1 (11.8,14.6)	15.8 (14.2,17.6)	13.8 (12.4,15.4)	10.9 (9.8,12.1)	8.4 (7.5,9.4)^b^	9.7 (8.7,10.8)			
Creatinine, mg/dL	W	0.91 (0.86,0.96)	0.77 (0.73,0.81)	0.75 (0.71,0.79)	0.70 (0.66,0.74)	0.69 (0.66,0.73)	0.78 (0.73,0.82)	**<0.01**	0.20	**<0.01**
	B	0.99 (0.91,1.07)	0.86 (0.79,0.93)^b^	0.78 (0.72,0.84)	0.72 (0.66,0.78)	0.66 (0.61,0.72)	0.77 (0.71,0.83)			
BUN/creatinine ratio	W	16 (15,17)	21 (19,22)	20 (18,21)	17 (15,18)	14 (13,15)	13 (12,14)	**<0.01**	**<0.01**	0.40
	B	13 (12,15)^b^	19 (17,21)	18 (16,20)	15 (14,17)	13 (11,14)	13 (11,14)			
Calcium, mg/dL	W	8.3 (8.2,8.4)	8.4 (8.3,8.5)	8.8 (8.6,8.9)	9.2 (9.0,9.3)	9.5 (9.3,9.6)	9.6 (9.5,9.7)	**<0.01**	**<0.01**	0.52
	B	8.6 (8.4,8.7)^b^	8.7 (8.6,8.9)^b^	9.1 (8.9,9.3)^b^	9.4 (9.2,9.6)	9.7 (9.5,9.9)	9.7 (9.6,9.9)			
TSP, g/dL	W	5.4 (5.3,5.5)	5.6 (5.4,5.7)	6 (5.8,6.1)	6.3 (6.2,6.4)	6.5 (6.3,6.6)	7.2 (7.1,7.3)	**<0.01**	**<0.01**	0.37
	B	6.0 (5.8,6.1)^b^	6 (5.9,6.2)^b^	6.4 (6.2,6.6)^b^	6.7 (6.5,6.8)^b^	6.8 (6.7,7.0)^b^	7.5 (7.3,7.7)^b^			
Albumin, g/dL	W	3.5 (3.4,3.6)	3.3 (3.2,3.4)	3.4 (3.3,3.5)	3.6 (3.5,3.7)	3.9 (3.8,4.0)	4.3 (4.2,4.4)	**<0.01**	0.43	**<0.01**
	B	3.7 (3.5,3.8)	3.4 (3.3,3.6)	3.5 (3.3,3.6)	3.6 (3.5,3.8)	3.8 (3.7,4.0)	4.2 (4.0,4.3)^b^			
Metabolism										
Cholesterol, mg/dL	W	148 (139,156)	159 (150,167)	169 (160,178)	178 (169,187)	195 (186,203)	190 (180,199)	**<0.01**	0.85	0.64
	B	153 (140,165)	158 (146,170)	166 (154,179)	178 (165,190)	190 (177,203)	187 (174,201)			
Triglycerides, mg/dL	W	104 (93,118)	151 (134,169)	139 (124,157)	161 (143,181)	182 (162,205)	147 (130,165)	**<0.01**	**<0.01**	**0.02**
	B	74 (63,88)^b^	126 (107,149)	116 (98,138)	120 (102,142)^b^	129 (109,153)^b^	102 (87,121)^b^			
Glucose, mg/dL	W	150 (143,158)	119 (113,125)	106 (102,111)	101 (96,105)	94 (90,98)	86 (82,90)	**<0.01**	0.57	**0.03**
	B	145 (135,156)	117 (108,127)	114 (107,121)	101 (95,108)	94 (88,100)	91 (85,97)			
Muscle										
Potassium, mEq/L	W	4.2 (4.3,4.1)	4.4 (4.4,4.3)	4.3 (4.4,4.2)	4.2 (4.3,4.1)	4.1 (4.2,4.1)	4.3 (4.3,4.2)	**<0.01**	**<0.01**	**0.02**
	B	4.2 (4.3,4.1)	4.6 (4.8,4.5)^b^	4.4 (4.5,4.3)	4.3 (4.4,4.1)	4.2 (4.4,4.1)	4.2 (4.4,4.1)			
Sodium, mEq/L	W	142 (141,143)	138 (137,138)	139 (138,140)	140 (139,141)	141 (141,142)	142 (141,142)	**<0.01**	**0.01**	0.60
	B	141 (140,142)	137 (136,138)	138 (137,139)	139 (138,140)	140 (139,141)	141 (141,142)			
Creatine kinase, u/L	W	1139 (940,1382)	214 (176,259)	111 (91,135)	64 (53,78)	43 (36,53)	88 (73,107)	**<0.01**	0.06	0.12
	B	12601 (945,1682)	235 (176,313)	122 (92,163)	103 (77,137)^b^	52 (39,69)	93 (70,124)			
Clotting										
Platelets, /mm^3^	W	161263 (148754,174823)	2415567 (222820,261869)	3443578 (317466,3735278)	2865945 (264365,3106934)	2656878 (245079,288029)	240320 (221680,260529)	**<0.01**	**<0.01**	0.70
	B	180065 (160039,202597)	273328 (242929,307530)	364934 (324082,410936)	319218 (283715,359162)	309035 (274665,347706)^b^	272639 (242317,306755)			
Prothrombin time, s	W	13.1 (12.7,13.5)	12.6 (12.2,13.0)	13 (12.6,13.4)	13.1 (12.7,13.5)	13.0 (12.6,13.4)	12.8 (12.4,13.1)	**<0.01**	0.08	0.67
	B	13.5 (13.0,14.1)	12.9 (12.4,13.5)	13.4 (12.9,14.0)	13.4 (12.8,14.0)	13.1 (12.5,13.7)	13 (12.5,13.6)			
Partial thromboplastin time, s	W	25.7 (24.5,26.9)	26.6 (25.4,27.9)	27.5 (26.2,28.8)	29 (27.7,30.4)	30.2 (28.9,31.7)	32.5 (31.1,34.1)	**<0.01**	0.14	0.44
	B	26.7 (25,28.4)	28.2 (26.5,30.0)	28.5 (26.7,30.4)	29.7 (27.9,31.6)	30 (28.2,31.9)	33.4 (31.3,35.5)			
Urine specific gravity	W	1.022 (1.021,1.024)	1.022 (1.021,1.024)	1.022 (1.02,1.023)	1.02 (1.019,1.022)	1.02 (1.019,1.022)	1.021 (1.02,1.022)	0.20	0.08	0.19
	B	1.024 (1.022,1.026)	1.021 (1.019,1.024)	1.022 (1.019,1.024)	1.022 (1.02,1.025)	1.022 (1.02,1.025)	1.022 (1.019,1.024)			
Urine pH	W	6.0 (5.8,6.1)	5.8 (5.6,5.9)	5.6 (5.5,5.8)	5.6 (5.5,5.8)	5.7 (5.5,5.8)	5.9 (5.7,6.0)	**<0.01**	0.55	0.36
	B	5.9 (5.6,6.1)	5.9 (5.6,6.2)	5.9 (5.6,6.2)	5.6 (5.3,5.8)	5.6 (5.4,5.9)	6 (5.7,6.3)			
Other										
Chloride, mEq/L	W	106 (105,107)	101 (100,102)	102 (101,102)	101 (101,102)	103 (102,103)	104 (104,105)	**<0.01**	**<0.01**	0.34
	B	105 (103,106)	100 (99,101)	101 (99,102)	101 (99,102)	102 (101,103)	104 (103,105)			
CO_2_, mmHg	W	27 (26,28)	27 (27,28)	27 (26,28)	27 (27,28)	27 (26,27)	26 (25,27)	**<0.01**	0.52	0.80
	B	27 (26,28)	28 (27,29)	27 (26,28)	27 (26,28)	27 (26,28)	26 (25,27)			
Inorganic phosphorus, mg/dL	W	2.6 (2.5,2.8)	4.0 (3.8,4.2)	4.3 (4.1,4.5)	4.5 (4.3,4.6)	4.5 (4.3,4.7)	3.8 (3.7,4.0)	**<0.01**	0.83	0.10
	B	2.9 (2.6,3.1)	4.1 (3.8,4.4)	4.2 (4,4.5.0)	4.3 (4.1,4.6)	4.4 (4.2,4.6)	3.7 (3.5,4.0)			
Uric acid, mg/dL	W	3.4 (3.1,3.7)	3.1 (2.8,3.4)	3.4 (3.1,3.7)	4 (3.7,4.3)	4.9 (4.5,5.4)	5.0 (4.6,5.5)	**<0.01**	0.69	**0.02**
	B	3.7 (3.2,4.1)	3.4 (3.0,3.9)	3.4 (3.0,3.9)	4 (3.5,4.6)	4.6 (4,5.2.0)	4.9 (4.3,5.5)			

Data are least-square means and 95% confidence intervals based on the linear mixed-effects model.

The bolded values indicate statistical significance or a trend.

*MCH* mean corpuscular hemoglobin, *MCHC* mean corpuscular hemoglobin concentration, *MCV* mean corpuscular volume, *AST* aspartate aminotransferase, *ALT* alanine transaminase, *Alk phosphatase* alkaline phosphatase, *BUN* blood urea nitrogen, *TSP* total serum protein.

^a^Statistical significance for the main effects of time, race, and their interaction.

^b^Statistically different between races based on post-hoc comparisons with Bonferroni adjustment.

^c^W: White individuals (*n* = 247), B: Black individuals (*n* = 69).

**Table 4:** Summary of racial differences in serological markers within the first year of spinal cord injury.

Outcome	SCI race effect?
Complete blood count	
White blood cells	Race and Time interaction, W < B initially and W > B at week 52
Neutrophils	Race and Time interaction, W > B at Week 52
Eosinophils	No effect of race
Monocytes	No effect of race
Lymphocytes	No effect of race
Basophils	Race and Time interaction, W < B initially and W > B at week 52
Hematocrit	Race and Time interaction, W > B at Week 52
Hemoglobin	Race and Time interaction, W > B at Week 52
Red blood cells	No effect of race
Mean corpuscular hemoglobin (MCH)	Race and Time interaction, W > B at baseline and week 52
Mean corpuscular hemoglobin concentration (MCHC)	No effect of race
Mean corpuscular volume (MCV)	Race and Time interaction, W > B at Week 52
Liver	
Aspartate transaminase (AST)	No effect of race
Alanine aminotransferase (ALT)	Main effect of race, W > B
Bilirubin	No effect of race
Alk phosphatase	No effect of race
Pancreas	
Amylase	Race and Time interaction, W < B at Weeks 8 and 52
Kidney	
Blood urea nitrogen (BUN)	Main effect of race, W > B
Creatinine	Race and Time interaction, the direction of difference changes over time, W < B at Week 2
BUN/creatinine ratio	Main effect of race, W > B
Calcium	Main effect of race, W < B
Total serum protein (TSP)	Main effect of race, W < B
Albumin	Race and Time interaction, W < B initially and W > B at week 52
Metabolism	
Cholesterol	No effect of race
Triglycerides	Race and Time interaction, W > B (but magnitude of difference varies over time)
Glucose	Race and Time interaction, but no significant differences
Muscle	
Potassium	Race and Time interaction, W < B at week 1
Sodium	Main effect of race, W > B
Creatine kinase	No effect of race
Clotting	
Platelets	Main effect of race, W < B
Prothrombin time	No effect of race
Partial thromboplastin time	No effect of race
Urine specific gravity	No effect of race
Urine pH	No effect of race
Other	
Chloride	Main effect of race, W > B
CO_2_	No effect of race
Inorganic phosphorus	No effect of race
Uric Acid	Race and Time interaction, W < B initially then W > B

Summary is based on linear mixed effect models with time, race, and their interaction as main variables of interest. The summary is intended to assist in interpreting Table 3 and should not be considered in isolation.

In addition to the main effect of time, significant main effects of race (no interaction effect) were observed for several markers. Specifically, White individuals had higher levels of ALT, BUN, BUN/Creatinine ratio, sodium, and chloride. Black individuals had higher levels of calcium, platelets, and total serum protein (TSP) (Table 3). Furthermore, White individuals tended to have higher MCHC (*p* = 0.06) and lower creatine kinase (*p* = 0.06).

Significant interaction effects, where the patterns of changes in serological markers differed over time, were observed for white blood cells, mature neutrophils, basophils, hematocrit, hemoglobin, MCH, MCV, Alk phosphatase, amylase, creatinine, albumin, triglycerides, glucose, potassium, and uric acid (Table 3). The patterns of the changes can be grouped as the following: 1) the values for white blood cells, hematocrit, hemoglobin, creatinine, Alk phosphatase, potassium, albumin, and uric acid were lower in White individuals at baseline or shortly after injury but subsequently became higher than Black individuals; 2) in contrast, glucose was initially higher in White individuals, then lowered over time to levels below Black individuals; 3) for several markers, the magnitude of the difference grew larger between races, such as mature neutrophils, lymphocytes, triglycerides, amylase, MCH, and MCV; 4) the direction of the difference changed twice over time (basophils).

For the following markers, serological marker values neither differed between races (i.e., no main effect of time), nor changed differently over time (i.e., no effect of time × race interaction): eosinophils, monocytes, red blood cell count, AST, bilirubin, cholesterol, partial thromboplastin time, urine pH, CO_2,_ and inorganic phosphorus.

## Discussion

Despite the well-documented differences in social determinants of health and disease risk, racial disparity outcomes in individuals with SCI have been rarely studied. This study aimed to address this gap and examine differences in serological markers between Black and White individuals with SCI across the first year of injury. There were time-dependent changes whereby most of markers fell outside of the normal range within the first few weeks of the injury and returned to normal ranges at one-year post-injury. This longitudinal progression has been previously reported and discussed in detail elsewhere [8]. Briefly, several variables, such as sex and injury severity were found to correlate with the changes in these markers, but the racial differences were not explored.

In our study, there were notable differences between Black and White individuals for multiple serological markers. In relation to muscle health, creatine kinase was higher for Black individuals. For the kidney, Black individuals had higher concentrations of calcium, BUN, and TSP, whilst White individuals had higher BUN/creatine ratio and albumin. In relation to metabolic health, triglycerides were higher for White individuals. Additionally, amylase concentrations were higher for Black individuals, while White individuals had higher ALT concentrations. For cell count markers, Black individuals had higher platelet counts and lymphocytes, whilst White individuals had higher hemoglobin, hematocrit, MCH, MCV, and neutrophils.

### Muscle health

Creatine kinase concentrations were slightly higher (~5%) in Black compared to White individuals (93 U/L vs. 87 U/L). Contrastingly, in a large-scale US NHANES study conducted in non-SCI general population, creatine kinase concentrations were almost two times higher in Black individuals than White individuals (Black: 140–160 U/L, White: 60–90 U/L) [14], with the difference not attributable to body composition. This comparative difference suggests that at one-year post-injury, Black patients with SCI have substantially lower creatine kinase compared to Black individuals in the non-SCI groups. A decreased creatine kinase is observed in several chronic conditions, such as prolonged bed rest, aging, and inflammation [15, 16]. Thus, it raises the need for further investigations into whether the racial disparities in creatine kinase in SCI could translate into longer-term health disparities.

### Kidney

There were racial differences in BUN (0.2–1.4 mg/dL higher for Black individuals) and BUN/creatinine ratio (0.8–2.7 lower for Black individuals). While these values were in normal ranges, higher concentrations of BUN are indicative of impaired kidney function [17]. In addition, creatinine concentrations were transiently higher in Black individuals only at one-week post-injury, with no further racial differences at other time-points. Higher creatinine concentrations for Black individuals are widely reported in non-SCI cohorts [18]. For example, NHANES data reports that mean creatinine levels are 1.25 (Black) and 1.16 (White) mg/dL for males, and 1.01 (Black) and 0.97 (White) mg/dL for females. In the current study, creatine concentrations are low compared to these national averages (0.78 and 0.77 mg/dL for White and Black individuals, respectively), and no racial differences were observed one year after injury. Lower creatinine concentrations can indicate muscle dystrophy, liver diseases, and poor nutritional status [19], and are associated with poor health outcomes in the critically ill [20]. The lack of racial differences indicates that Black patients with SCI had further reduced creatinine concentrations. Future studies are needed to understand the cause and health implications of the lower creatinine concentrations for Black individuals within the first year of injury.

Black individuals had higher concentrations of calcium compared to White individuals at each measured time point (difference: 0.16–33 mg/dL). Consistent with this, previous research has shown that those with a higher serum calcium concentration tend to be Black compared to White [21]. Racial differences in calcium homeostasis have been consistently reported in non-SCI cohorts, with Black individuals reported to have higher bone mineral density [22], increased calcium absorption [23], and lower urine calcium excretion [24] compared to White individuals. Serum calcium has been reported to have a U-shaped association with all-cause mortality, with race having a modifying effect on the association [25]. When calcium was ≥8.8 mg/dL, African American’s had lower risk of mortality, and when calcium was <8.8 mg/dL, African Americans had a higher mortality rate risk. On the group level, in this study, participants’ calcium concentrations rose to 8.8 mg/dL by week two for both races. Although race may have a protective effect on mortality in African American individuals, whether the slightly elevated calcium levels could counteract the protective effect is unknown in SCI.

Black individuals had higher concentrations of TSP (difference: 0.30–0.52 g/dL across different time points) and lower albumin concentrations (difference: 0.16 g/dL at one-year post-injury) compared to White individuals. This is consistent with previous studies in non-SCI cohorts [10, 26, 27]. Albumin is a main component of the total protein, and a lower albumin and higher TSP in our cohort of Black individuals indicates an elevated ratio of globulin fraction. A higher globulin fraction could indicate several disease conditions, such as malnutrition, acute inflammation, and liver disorders [28]. However, there is limited research evaluating whether such racial differences could result from a higher prevalence of these disease conditions or genetic variations [26].

### Metabolism

Triglycerides (fasting and non-fasting) are regarded as a traditional cardiometabolic risk factor that is strongly associated with type-2 diabetes and heart disease risk [29]. There was a clear and consistent racial difference in triglycerides across the first year of injury. Specifically, mean triglyceride concentrations were greater for White compared to Black individuals, equating to a 44.4 mg/dL difference at one year post-injury, while the concentrations for both are within the normal range (<150 mg/dL). This effect has been consistently reported in non-SCI populations [30, 31]. Triglyceride concentrations have been reported to be 24.8 mg/dL (males) and 33.3 mg/dL (females) higher for Non-Hispanic Whites (*n* = 2427) compared to African Americans (*n* = 1519) [31]. Furthermore, this is consistent with the lower prevalence of cardiometabolic syndrome in Black compared to White individuals based on the US NHANES survey [32], which is likely to persist in individuals with SCI. In non-SCI cohorts, higher concentrations of triglycerides have been associated with an increased risk of cardiovascular disease, with the effect dependent on race. The present study showed no significant differences between races for glucose or cholesterol. Given that a plethora of risk factors collectively contribute to the development of cardiovascular disease, future evaluations are needed to understand if racial disparities exist in cardiometabolic diseases in SCI.

### Pancreas

Amylase concentrations were greater for Black compared to White individuals, equating to a 22.3 U/L difference at one year post-injury. This difference was also evident 8 weeks post-injury (19.4 U/L). These results are consistent with studies from non-SCI populations [33, 34]. For example, serum amylase concentrations have been reported to be 21.0 U/L higher for Black (*n* = 123) compared to White individuals (*n* = 125) [33]. In non-SCI groups, several studies have suggested the use of amylase as a biomarker for metabolic disorders. Specifically, individuals with metabolic disorders, such as type 2 diabetes, obesity, and multiple sclerosis have been reported to have lower serum amylase concentrations compared to healthy individuals (metabolic disorder: ~44–90 U/L vs healthy: ~150 U/L) [35–37]. However, no research has evaluated whether the elevated amylase concentrations in Black individuals could put them at a higher risk of metabolic disorders. Interestingly, regardless of the racial differences, both groups’ amylase concentrations fell within the range seen in individuals with metabolic disorders. This observation underscores the fact that SCI-induced physiological (e.g., loss of lean muscle mass) and lifestyle (e.g., increased fat mass, physical deconditioning) changes often increase the risk of metabolic impairment [38, 39].

### Liver

There was also a racial difference in ALT, a marker of liver damage. White individuals had higher ALT concentrations compared to Black individuals, with a modest difference of 2.1 U/L observed at one year post-injury. White individuals (20.6 U/L) had mean concentrations above the recommended upper limit (20 U/L), while Black individuals (18.5 U/L) remained below this upper limit. This difference has been reported in a large non-SCI cohort (*n* = 6179) with a combined group of Asian American/White/Other individuals having higher ALT values (2.2 U/L) than African Americans [40]. Therefore, it has been suggested that race-specific normal ranges for ALT should be developed to ensure Black individuals are appropriately screened for liver disease [40]. Clinicians treating Black SCI patients with ALT concentrations close to the upper limit should carefully consider further evaluation for liver diseases such as cirrhosis and non-alcoholic fatty liver disease [9]. Furthermore, physicians specializing in SCI need to be cognizant of these differences when managing patients, and researchers should incorporate these reference intervals when screening potential participants for clinical trials in this population.

### Cell count

At one year post-injury, White individuals had higher concentrations of hemoglobin (0.87 g/dL), hematocrit (2.45%), MCH (1.16 pg/cell), and MCV (3.21 µm^3^) compared to Black individuals. These differences are consistent with non-SCI literature [41–44]. Similarly, at one year post-injury, Black individuals had higher concentrations of lymphocytes (5.6%) compared to White individuals, while White individuals had higher concentrations of (7.5%) neutrophils compared to Black individuals [9]. Although all markers were within uniform reference ranges, different reference ranges for races have been proposed for these markers [9]. Physicians specializing in SCI should be aware of these in the treatment of patients, whilst researchers should use these reference intervals when screening potential participants for clinical trials.

Black individuals had higher platelet counts than White (~12% difference) at one year post-injury. A comparable magnitude of difference has been reported in non-SCI populations after controlling for age and sex [45]. Increased platelet counts have been linked with various disease risks, such as anemia, cancer, homocysteine, and atherosclerotic risk [45]. Whether such racial differences may contribute to an increased risk of chronic disease or illness for Black individuals with SCI needs to be further explored.

### Future directions

Achieving racial health equity is a morally imperative task, requiring enormous, concerted efforts from researchers, policymakers, and healthcare providers. Our study provided initial evidence of racial differences in commonly measured serological markers in the first year of spinal cord injury (SCI). However, whether these differences translate into long-term disparities in health outcomes needs evaluation, necessitating larger longitudinal cohort studies. Furthermore, future research should investigate the underlying mechanisms contributing to the observed racial differences in serological markers, including socioeconomic factors, access to healthcare, and cultural influences. Importantly, our study observed racial differences in several markers consistent with those in the general non-SCI population, where they were attributed to normal biological diversity (e.g., blood cells) [9, 46]. For these markers, it remains unclear whether such differences also indicate normal biological diversity in SCI. Addressing these questions is crucial for developing social, policy, and healthcare changes to provide tailored healthcare management strategies, implementing social health equity initiatives, and employing precision medicine approaches to achieve health equity among patients of diverse racial backgrounds.

### Limitations

The main limitation of this study is the retrospective nature of the data utilized, with the original samples collected 25–30 years prior (1992–1997). This limits the translation of the findings to the current clinical context. For example, it was not possible to include other racial groups (e.g., Asian, Hispanic) due to a lack of other races in the participant cohort. Moreover, the administration of steroids, which could influence the serological markers, was a common practice during this time frame. Additionally, the original study excluded patients with pre-existing conditions (e.g., lung, liver, gastrointestinal, or kidney disease). As a result, the findings of the study may not be generalizable to the broader SCI population. Furthermore, only individuals who survived the first year and completed the 1-year follow-up visit were included, which may introduce a survivorship bias. We did not control for some factors (e.g., socioeconomic status, participation in physical therapy, and medications) that are known to affect health status [47]. Finally, despite having a relatively large sample size for an SCI study, more data are needed to detect minor racial differences in serological markers. Therefore, it’s possible that specific serological markers that showed no significant differences between racial groups in this study could still be affected by race.

## Conclusions

Several notable racial differences in serological markers, including triglycerides, amylase, ALT, calcium, BUN, and platelet count, exist across the first year of injury following an SCI. These differences underscore the importance of considering race as a critical determinant of physiological responses and health outcomes post SCI. Future research is warranted to investigate the cause of such disparities and their implications for long-term health in SCI. By understanding and exploring these differences, stakeholders, such as healthcare providers and policymakers, can develop tailored care and social programs that address the specific needs of different racial groups, thereby improving health equity.

## Data Availability

Anonymized data used in this study will be made available upon request to the corresponding author and in compliance with the General Data Protection Regulation (EU GDPR).
